# Paving the Path to Prevent Peripartum Hysterectomies: Risk Stratification in Placenta Accreta Spectrum

**DOI:** 10.34763/jmotherandchild.20252901.d-25-00011

**Published:** 2025-08-16

**Authors:** Ashmeet Kaur, Kalpana Mangal, Ankita Kumari Sharma, Mahi Gupta, Aditi Bansal, Pritosh Yadav

**Affiliations:** Department of Pathology, SMS Medical College, Jaipur, Rajasthan, India; Department of Pathology, Geetanjali Medical College and Hospital, Udaipur, India; Department of Obstetrics and Gynecology, Zanana Hospital, SMS Medical College and Hospital, Jaipur, India

**Keywords:** Placenta Accreta Spectrum, Peripartum Hysterectomy, Risk Factors, Caesarean Section, Placenta Praevia, IVF, Uterine Surgeries

## Abstract

**Background:**

Placenta Accreta Spectrum (PAS) is a life-threatening obstetric condition with increasing incidence due to rising caesarean deliveries and assisted reproductive technologies.

Our objective was to determine PAS incidence, identify risk factors, and develop a clinically relevant risk stratification model.

**Material and methods:**

A retrospective study of 85 PAS cases from 9,088 deliveries (September 2023 to September 2024, SMS Medical College, Jaipur) analysed clinical and histopathological data, including placenta praevia, multiparity, prior caesarean sections, uterine surgeries, and IVF. Cases with spontaneous placental separation were excluded.

**Results:**

PAS incidence was 0.94%. Placenta accreta, increta, and percreta were found in 35.3%, 34.1%, and 30.6% of cases, respectively. Significant risk factors included multiparity (82.4%, p < 0.001), prior caesarean sections (88.2%, p < 0.05), placenta praevia (70.6%, p = 0.002), uterine surgeries (21.17%, p < 0.05), and IVF (7.1%, p < 0.05). A PAS risk model integrating clinical predictors and region-specific weighted scoring was developed for early identification.

**Conclusion:**

PAS is a significant obstetric challenge. Identified risk factors include multiparity, prior caesarean sections, placenta praevia, uterine surgeries, and IVF. Early detection and structured referral pathways are critical for reducing maternal morbidity. This study bridges the gap between region-specific data and global PAS trends, offering a tailored, evidence-based risk stratification model for improved maternal care in resource-limited settings.

## Introduction

First formally described by Irving in 1937, placenta accreta is a life-threatening condition where the placenta fails to detach from the uterine wall after delivery [1]. Historical references to similar conditions date back to the late 1500s, with Plater documenting a case from 88 AD. Once rare, its prevalence increased from 1 in 7,000 to 1 in 533 deliveries by the early 2000s, due to rising caesarean rates and assisted reproductive technologies (ART) [2].

PAS refers to a spectrum of conditions in which the placenta abnormally invades the uterine wall, potentially leading to severe complications such as massive haemorrhage. The International Federation of Gynaecology and Obstetrics (FIGO) defines PAS as ranging from superficial placental attachment (placenta accreta) to deep invasion into surrounding organs (placenta percreta) [3]. Multidisciplinary management, as emphasized by FIGO, the Royal College of Obstetricians and Gynaecologists (RCOG), and the American College of Obstetricians and Gynecologists (ACOG), is critical for optimizing outcomes [3,4,5].

The pathogenesis of PAS is linked to defective decidualization at the endometrial-myometrial interface, often in areas previously damaged by uterine surgeries. This damage results in an insufficient decidual layer, allowing unchecked intrusion of cytotrophoblasts into the myometrium. Histopathological studies have revealed trophoblast invasion without an intervening decidual layer, trophoblast inclusions, and reduced natural killer cells [6]. Additionally, caesarean-scar pregnancy, a precursor to PAS, occurs when a blastocyst implants on a uterine scar, contributing to abnormal placental development. Reduced vascularization in caesarean scar tissue creates a hypoxic environment that may attract blastocyst implantation and promote abnormal placental invasion [6]. Advanced maternal age and infertility treatments, such as IVF, compound these risks [7].

In India, caesarean deliveries have increased substantially, reflecting changing obstetric practices and a rise in institutional deliveries. For instance, In Rajasthan, caesarean rates rose from 9.0% in 2015–16 to 14.2% in 2019–20, correlating with increased placenta praevia rates (0.3% to 1.8%) [8]. Routine third-trimester ultrasounds are crucial for early detection, enabling timely referrals to tertiary care centres and reducing complications [9].

Post-delivery histopathological examination plays a vital role in confirming PAS diagnoses. Biopsy samples from hysterectomy specimens or placental tissues can verify the extent of trophoblastic invasion into the myometrium and differentiate between accreta, increta, and percreta. Studies emphasize that post-delivery histological analysis confirms the diagnosis and provides critical insights into the severity of placental invasion, guiding postoperative management and future pregnancy planning [6,10,11].

The NFHS data underscores the need to expand access to quality obstetric care, promote routine ultrasonography, and integrate biopsy protocols for postpartum diagnosis of conditions like PAS and placenta praevia. Addressing these issues is crucial for improving maternal and neonatal outcomes, particularly in regions with limited healthcare infrastructure.

While numerous studies have explored PAS risk factors, few have attempted to develop a scoring model incorporating both obstetric history and imaging markers. This study aims to address that gap, particularly in the Indian context, where region-specific risk stratification is lacking. To determine the incidence of PAS in a tertiary care setting, it evaluates maternal outcomes in peripartum hysterectomy cases, and develops a risk stratification model incorporating key clinical and imaging parameters.

## Materials and methods

### Study design

This study follows a retrospective, cross-sectional design analysing cases of obstetric hysterectomy due to Placenta Accreta Spectrum (PAS). Data was collected from hospital records and histopathological reports at SMS Medical College, Jaipur, from September 2023 to September 2024.

### Ethical approval

All procedures performed in this study were in accordance with the ethical standards of the institutional and/or national research committee and with the 1964 Helsinki Declaration and its later amendments or comparable ethical standards. As this was a retrospective observational study, ethical approval was waived by the Institutional Review Board of SMS Medical College. Patient confidentiality was maintained throughout. This study follows STROBE guidelines for observational research.

### Inclusion and exclusion criteria

All cases of histopathologically-confirmed PAS were included. Cases with spontaneous placental separation, uterine pathologies unrelated to PAS, or incomplete records were excluded.

### Data collection

The study data were retrospectively retrieved from electronic medical records and histopathological reports maintained by the Department of Pathology, SMS Medical College. Clinical parameters — including placenta praevia, multiparity, prior caesarean sections, uterine surgeries, and IVF, along with prenatal ultrasound and Doppler findings — were extracted and systematically correlated with intraoperative and histopathological assessments.

### Histopathological evaluation

Grossing included the number of specimens taken from the placental site and documentation of the depth of invasion. To reduce bias, cases were reviewed independently by two pathologists, and missing data were addressed through hospital archives where available. Hematoxylin and eosin-stained sections confirmed diagnoses of accreta, increta, or percreta.

### Statistical analysis

Chi-square tests and logistic regression were utilized to retrospectively evaluate risk factors associated with PAS. Adjusted odds ratios (ORs) with 95% confidence intervals (CIs) were computed to quantify risk associations. Statistical analysis was conducted using PSPP, with p < 0.05 considered statistically significant. To develop a PAS risk stratification model, weighted risk scores were derived from ORs of individual risk factors. These ORs were normalized and incorporated into a structured point-based system, enhancing clinical applicability. The final score represents the cumulative influence of multiple risk factors, aiding in effective PAS risk assessment and stratification.

## Results

### Patient demographics

The patient cohort ranged in age from 23 to 42 years (mean: 32), with 94.1% of cases occurring beyond 34 weeks of gestation.

### Clinical presentation

Antenatal bleeding (41.2%) was the most frequent symptom, followed by postpartum haemorrhage (17.6%), preterm labour (4.7%), and hypertension (2.4%). Table 1 summarizes clinical presentation and associated intraoperative complications.

**Table 1.** The table provides a detailed comparison of incidence rates, key risk factors and clinical outcomes between PAS and non-PAS cases

**S. No.**	**Category**	**Parameter**	**No. of PAS Cases**	**No. of PAS Cases (%)**	**No. of Non-PAS Cases**	**No. of Non-PAS Cases (%)**	***P*-Value**
1.	Total cases	9088	85	0.93%	9003		0.53
2.	Clinical presentation	Antenatal bleeding	35	41.2%	3707	41.1%	0.06
		Postpartum haemorrhage	15	17.6%	1482	16.46%	0.1
		Hypertension	2	2.4%	211	2.3%	0.3
		Preterm labor	4	4.7%	317	3.5%	0.25
3.	Risk factors	Multiparity	70	82.4%	5000	55.5%	0.0001
	Previous caesarean sections						
		1 Previous	35	41.2%	3500	38.9%	0.05
		2 Previous	25	29.4%	2000	22.2%	0.0001
		3 + Previous	15	17.6%	800	8.9%	0.002
		Placenta praevia	60	70.6%	2000	22.2%	0.002
4.	History of uterine Surgeries	Myomectomy	10	11.8%	500	5.6%	0.005
		Other surgeries	8	9.4%	400	4.4%	0.03
5.	History of IVF	Present	6	7.1%	200	2.2%	0.05
6.	Ultrasound & doppler findings	Loss of retroplacental Zone	50	58.8%	1500	16.7%	0.001
		Placental lacunae	40	47.1%	1200	13.3%	0.002
		Hypervascularity on Doppler	55	64.7%	1800	20.0%	0.0005
7.	Maternal Outcomes	≥ 4 Units transfused	30	35.3%	3000	33.3%	0.2
		Hysterectomy required	40	47.1%	50	0.6%	0.0001
		ICU admission	8	9.4%	741	8.2%	0.3
		Mortality	1	1.2%	105	1.16%	0.5
8.	Neonatal Outcomes	Preterm Birth (< 37 weeks)	60	70.6%	4000	44.4%	0.0008
		NICU Admission	50	58.8%	3500	38.9%	0.0015

### Types of abnormal placentation

**Figure 1.**
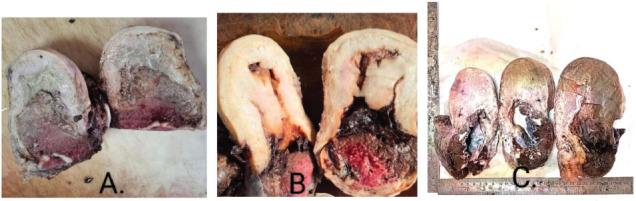
Gross morphology of abnormal placentation. **A.** Placenta increta: Cross-section showing invasion of placental tissue into the myometrium. **B.** Placenta accreta: Cross-section demonstrating placental adherence to the myometrium without significant invasion. **C.** Placenta percreta: Cross-section revealing placental invasion through the uterine wall, potentially involving serosa or adjacent organs.

Placenta accreta was diagnosed in 35.3% of cases. Placenta increta and placenta percreta accounted for 34.1% and 30.6% of the cases, respectively. Gross morphological features of abnormal placentation, as shown in Fig. 1, displayed varying depths of invasion, ranging from accreta to percreta.

### Histopathological findings

**Figure 2.**
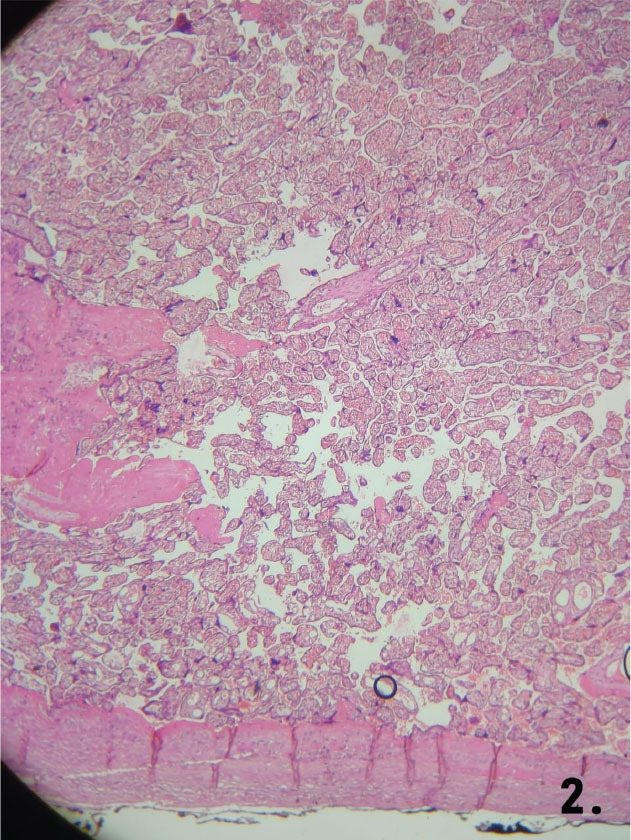
Histological section showing placenta accreta with chorionic villi directly attached to the myometrium, lacking the intervening decidual layer (HE 10 ×).

**Figure 3.**
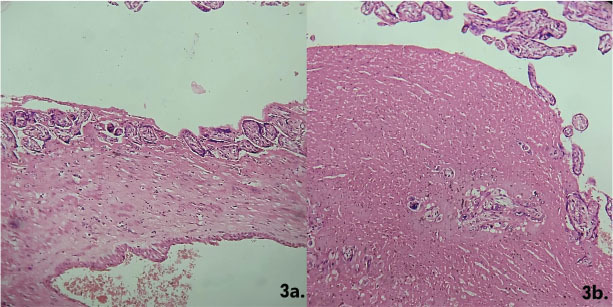
**a.** Histological section showing placenta increta with chorionic villi invading the myometrial tissue, indicating deeper penetration. (HE 40 ×) **b.** Histological section showing deep chorionic villi invasion of the myometrial tissue, which disrupts its architecture. (HE 40 ×).

**Figure 4.**
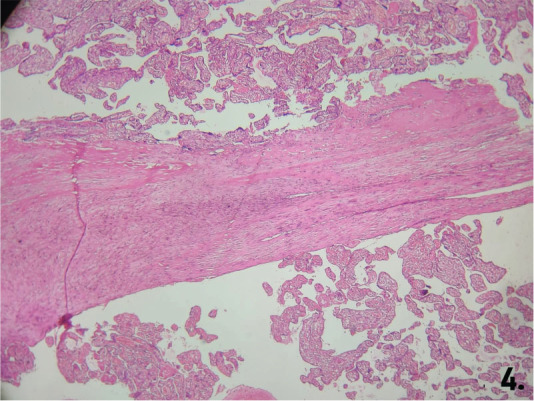
Histological section showing placenta percreta with chorionic villi invading through the entire thickness of the myometrium, reaching the uterine serosa. (HE 10 ×).

Placenta accreta cases showed chorionic villi directly attached to the myometrium without a decidual layer (Fig. 2). Placenta increta cases demonstrated villi invading the myometrium, disrupting its architecture (Figs. 3a and 3b). Placenta percreta cases exhibited villi invading the entire myometrium, extending to the uterine serosa (Fig. 4).

### Risk factors

Multiparity, previous caesarean sections, and placenta praevia were the most commonly observed risk factors in this study.

Multiparity was observed in 82.4% of cases, showing a strong statistical association (p = 0.0001). The risk of PAS significantly increased with parity, with an odds ratio of 3.8 (95% CI, 2.17–6.65).

Previous caesarean sections were noted in 88.2% of cases, with PAS risk increasing substantially as the number of caesareans rose. For patients with three or more caesarean deliveries, the PAS incidence reached 17.6%, showing a statistically significant association (p = 0.002) and an odds ratio of 6.5. Each successive caesarean added to the cumulative risk; this drives home the fact that repeated surgical procedures compromise uterine integrity and elevate PAS susceptibility.

**Figure 5.**
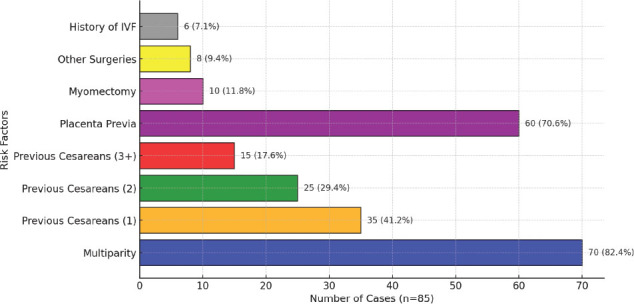
Distribution of key risk factors in Placenta Accreta Spectrum (PAS) cases.

Placenta praevia was present in 70.6% of PAS cases, with a strong correlation evident in both univariate and multivariate analyses. The Chi-square test confirmed this association as statistically significant (p = 0.002), with a relative risk of 3.18 (95% CI, 2.76–3.66). Multiparity and placenta praevia emerged as the most prominent contributors to PAS risk, underscoring the importance of meticulous antenatal monitoring, particularly in high-risk pregnancies. Fig. 5 illustrates the distribution of these critical risk factors.

### Ultrasound and Doppler findings

**Figure 6.**
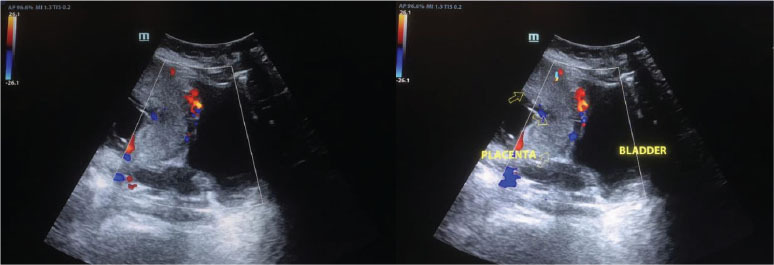
Colour Doppler ultrasound images showing protrusion of placental tissues beyond the outer confines of the uterine myometrium, with thinning of the uterine serosa-bladder wall complex and increased vascularity between the serosa and the bladder.

Prenatal imaging was vital for PAS diagnosis, detecting retroplacental zone loss in 58.8% of cases (p = 0.001), placental lacunae in 47.1% (p = 0.002), and Doppler hypervascularity in 64.7% (p = 0.0005). The colour Doppler images in Fig. 6 show placental tissue protrusion beyond the uterine myometrium, with serosa-bladder wall thinning and increased vascularity.

### Maternal outcomes

In PAS cases, 9.4% of patients required ICU admission, and hysterectomy was performed in 47.1% of deliveries (p = 0.0001). Additionally, 35.3% needed ≥ 4 units of blood transfusion (p = 0.2), highlighting the significant maternal morbidity associated with PAS.

### Neonatal outcomes

PAS had a notable impact on neonatal outcomes, with 70.6% of cases leading to preterm birth before 37 weeks (p = 0.0008). Furthermore, 58.8% of neonates required NICU admission (p = 0.0015), underscoring the substantial perinatal complications associated with PAS.

### Risk scoring model

The PAS risk scoring model integrates clinical, surgical, and imaging factors to stratify patients from low to high risk (0 to ≥30 points). This weighted risk assessment incorporates statistically significant factors from 85 retrospective cases to improve the prediction of high-risk pregnancies.

**Figure 7.**
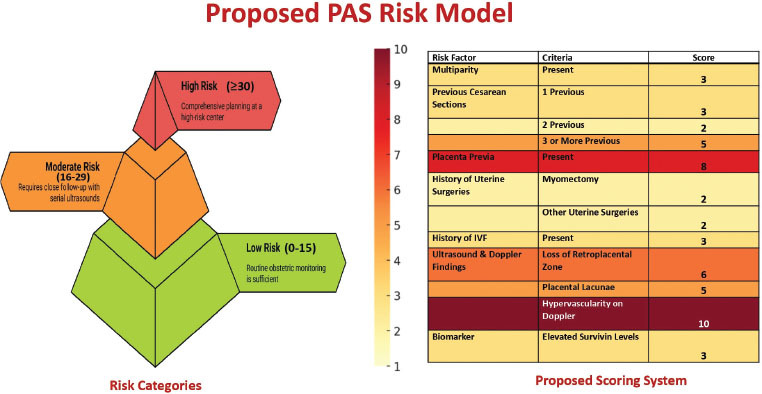
Proposed PAS risk model, with comprehensive scoring and risk categorisation system.

Fig. 7 shows a colour-coded tiered system with risk categories defined by cumulative scores from clinical and imaging factors. Multiparity (OR = 3.8, 3 points), previous caesarean sections, weighted by number (e.g., 3+ caesareans: OR = 6.5, 5 points), and placenta praevia (OR = 9.0, 8 points) were key contributors. Uterine surgeries (myomectomy: OR = 3.0, 4 points; other surgeries: OR = 2.2, 2 points), IVF (OR = 3.4, 3 points), and imaging markers (retroplacental zone loss: OR = 7.5, 6 points; lacunae: OR = 5.5, 5 points; Doppler hypervascularity: OR = 12.0, 10 points) were included. Survivin levels, though not analysed here, were integrated for their emerging role in PAS. The scoring range (0 to ≥ 49) stratifies patients into low (0 to 15), moderate (16–29), and high (≥ 30) risk categories, offering a structured PAS prediction and intervention framework.

## Discussion

The observed incidence of placenta accreta spectrum (PAS) in our cohort was 0.94% (85 out of 9,088 deliveries); this is consistent with the higher end of the globally reported incidence rates, which range from 0.1% to 2.3%. Caesarean deliveries and prior uterine surgeries continue to be recognized as major contributing factors worldwide [12,13,14]. A 2019 meta-analysis reported a pooled prevalence of 0.17% (95% CI: 0.14–0.19%), though variations are influenced by differences in obstetric practices, diagnostic methods, and referral systems [12]. The elevated incidence in our study likely reflects region-specific challenges — including delayed referrals, limited access to prenatal screening, and a high rate of emergency presentations — which are often under-reported in larger epidemiological studies.

Regional disparities further highlight the role of obstetric practices in shaping PAS burden. For instance, a 2022 Chinese multi-centre study by Ming Y et al. [13] reported a prevalence of 2.20%, while El Gelany et al. [14] documented 0.91%, both linked to elevated caesarean rates and multiparity [13,14]. This variability highlights how surgical trends, diagnostic capabilities, and access to prenatal care influence PAS rates across different populations.

In India, the increasing incidence of PAS corresponds with an increase in caesarean deliveries. Rajasthan's caesarean rate surged from 9.0% in 2015–16 to 14.2% in 2019–21 according to NFHS-5 data [8], reflecting trends observed in high-incidence regions globally. Studies by Mittal et al. [3], Jauniaux et al. [12], and Gelany et al. [14] demonstrate that unregulated caesarean practices heighten PAS risk; this underscores the need for evidence-based surgical decisions. These findings support standardized risk stratification, efficient referral systems, and multidisciplinary care models to reduce maternal morbidity in high-risk obstetric populations.

Our results reaffirm multiparity (82.4%) and placenta praevia (70.6%) as predominant PAS risk factors, with the latter's significant association (p < 0.002) consistent with global patterns. The escalating risk with prior caesarean sections — 17.6% after ≥ 3 surgeries — emphasizes the importance of curbing unnecessary repeat procedures. Less-common contributors, such as uterine surgeries (9.4%) and IVF (7.1%), highlight the cumulative impact of surgical and reproductive interventions on uterine integrity. These findings align with established literature (Varlas et al. [15], Silver et al. [9]) and stress the importance of obstetric history in PAS risk stratification. Our data advocate targeted monitoring in high-risk subgroups to optimize resource use and prenatal care.

The observed ultrasound markers in our study — loss of the retroplacental zone (58.8%), placental lacunae (47.1%), and hypervascularity on Doppler (64.7%) — align closely with the standardized PAS detection criteria proposed by Adu-Bredu et al. [16]. Their three-step method highlights these imaging features as essential for risk stratification, particularly in resource-limited settings; this is consistent with our findings on the utility of basic ultrasound protocols for early PAS diagnosis.

The high ICU admission rate (9.4%) and hysterectomy incidence (47.1% of PAS deliveries, p = 0.0001) underscore the life-threatening nature of PAS. Effective management and reduction of maternal morbidity requires comprehensive surgical planning, timely access to blood products, and critical care resources. Although the need for ≥ 4 units of blood transfusion (35.3%) was not statistically significant (p = 0.2), it indicates a clinically relevant haemorrhagic risk, which necessitates preoperative blood bank coordination and multidisciplinary surgical planning to prevent adverse outcomes.

PAS significantly affects perinatal health, occurring in 70.6% of preterm births (p = 0.0008) and 58.8% of NICU admissions (p = 0.0015), often due to the use of iatrogenic preterm delivery to safeguard maternal well-being. Beyond delivery, PAS management poses long-term maternal health risks — including secondary infertility and adhesion-related complications — that warrant further study. The high frequency of emergency interventions highlights the need for structured referral pathways to ensure timely risk identification and planned management. Delayed referrals and emergency procedures exacerbate complications, underscoring the importance of early triage and risk-based transfer protocols. The association between PAS and preterm complications (p = 0.0008) underscores the necessity for individualized delivery timing and specialized neonatal care teams to mitigate perinatal morbidity.

A key strength of this study is its PAS-specific risk stratification model, tailored for high-risk obstetric populations. Unlike traditional categorical assessments, this model employs weighted scoring that reflects real-world clinical urgency, quantifying risk severity rather than merely identifying risk factors. Prior caesarean sections are assigned incremental point levels (2–5 points) based on the number of surgeries, which highlights their dose-dependent impact on PAS risk. Placenta praevia (8 points) and multiparity (3 points) are also weighted according to their prevalence and mechanistic role in abnormal placentation.

This model also stands out due to its inclusion of understudied risk factors such as uterine surgeries (2 points) and IVF (3 points). This is particularly relevant in India, where ART use and non-caesarean uterine interventions are rising. Including emerging biomarkers like serum Survivin levels, which are associated with abnormal placentation, further enhance the model's predictive potential. Future studies should investigate multi-marker panels, combining Survivin with angiogenic factors such as PIGF-1 and sFlt-1 to refine PAS risk assessment. Incorporating biomarker analysis with imaging and clinical data offers a comprehensive diagnostic approach, bridging gaps in resource-limited settings where advanced imaging is often unavailable, but basic ultrasound is accessible.

The PAS risk stratification framework categorizes patients into actionable risk groups: low risk (0–15 points) for routine monitoring; moderate risk (16–29 points), requiring serial ultrasound follow-up; and high risk (≥ 30 points), necessitating multidisciplinary planning and delivery at a specialized centre. This model enhances clinical workflows, especially in resource-limited settings, by enabling early triage and structured referrals, potentially reducing emergency interventions, and improving preparedness. Integrating this approach into standard obstetric care protocols can enhance consistency in PAS risk assessment, triage, and management.

This stratification aligns with global efforts for standardized PAS protocols while addressing region-specific challenges such as delayed referrals and fragmented care systems. Our findings highlight the detrimental impact of late referrals on PAS outcomes, emphasizing the importance of early triage and structured transfer pathways in mitigating maternal and neonatal risks. Unlike prior models, our framework transcends simple risk aggregation by providing a dynamic, context-sensitive tool for proactive obstetric care.

The novelty of this model lies in its weighted, context-specific design, which enhances accuracy without compromising practicality. Validating this model across diverse populations will be essential to establishing its role in reducing PAS-related morbidity, particularly in regions with rising caesarean rates and ART utilization. This study bridges the critical gap between region-specific obstetric data and global PAS trends by integrating local clinical predictors into an evidence-based risk-stratification model. It improves the understanding of PAS in resource-limited settings and offers a tailored approach for enhancing maternal care through early diagnosis and intervention. Comparative analysis with existing PAS models could further highlight the strengths of this scoring approach. Prospective multi-centre studies will be vital for confirming its broader clinical utility, and its adaptability makes it a promising tool for routine obstetric workflows.

## Conclusion

In conclusion, this study presents a region-specific PAS risk stratification model that integrates clinical and imaging parameters to enhance early diagnosis and intervention. It underscores late referral as a significant factor contributing to adverse maternal outcomes and advocates for establishing structured referral pathways in high-risk pregnancies. Our findings aim to improve PAS risk prediction and management, especially in regions with rising caesarean rates and evolving reproductive practices. Future studies should focus on refining this model, validating its predictive accuracy, and exploring preventive strategies to reduce PAS burden and improve maternal care, particularly in resource-limited settings.
